# An Accurate GPS-IMU/DR Data Fusion Method for Driverless Car Based on a Set of Predictive Models and Grid Constraints

**DOI:** 10.3390/s16030280

**Published:** 2016-02-24

**Authors:** Shiyao Wang, Zhidong Deng, Gang Yin

**Affiliations:** 1 State Key Laboratory of Intelligent Technology and Systems, Tsinghua National Laboratory for Information Science and Technology, Department of Computer Science, Tsinghua University, Beijing 100084, China; sy-wang14@mails.tsinghua.edu.cn (S.W.); michael@tsinghua.edu.cn (Z.D.); 2 State Key Laboratory of Coal Mine Disaster Dynamics and Control, College of Resources and Environmental Science, Chongqing University, Chongqing 400030, China

**Keywords:** local navigation, GPS-IMU/DR integrated navigation, multimodal data fusion, maximum likelihood estimation, driverless car

## Abstract

A high-performance differential global positioning system (GPS)  receiver with real time kinematics provides absolute localization for driverless cars. However, it is not only susceptible to multipath effect but also unable to effectively fulfill precise error correction in a wide range of driving areas. This paper proposes an accurate GPS–inertial measurement unit (IMU)/dead reckoning (DR) data fusion method based on a set of predictive models and occupancy grid constraints. First, we employ a set of autoregressive and moving average (ARMA) equations that have different structural parameters to build maximum likelihood models of raw navigation. Second, both grid constraints and spatial consensus checks on all predictive results and current measurements are required to have removal of outliers. Navigation data that satisfy stationary stochastic process are further fused to achieve accurate localization results. Third, the standard deviation of multimodal data fusion can be pre-specified by grid size. Finally, we perform a lot of field tests on a diversity of real urban scenarios. The experimental results demonstrate that the method can significantly smooth small jumps in bias and considerably reduce accumulated position errors due to DR. With low computational complexity, the position accuracy of our method surpasses existing state-of-the-arts on the same dataset and the new data fusion method is practically applied in our driverless car.

## 1. Introduction

Autonomous navigation is one of the most key technologies for driverless cars. Accurate positioning and orientation estimation of vehicles is generally regarded as the basis of many sophisticated modules such as environmental perception, path planning, and autonomous decision-making of driverless cars under complex urban scenarios. Different from stand-alone GPS that is increasingly a popular navigation system, an enhanced differential GPS (DGPS) receiver with phase carrier signal measurements may run in operating modes of real time kinematics (RTK–DGPS), which has the highest absolute position accuracy of up to a few centimeters. In DGPS, mobile GPS device continuously receives correction data from ground-based reference station over transmitter of shorter range, aiming to compensate location inaccuracies [1]. DGPS systems operating under complicated urban scenarios, however, occasionally lose broadcast signals and probably acquire inaccurate localization data due to many unpredictable factors such as buildings’ occlusion, signal attenuation, and a diversity of electronic interference. In general, it works well in a limited range in terms of pseudorange correction principle. In addition, atmospheric visibility of satellite, potential environmental effects, and multipath may have negative impact on precision and reliability of GPS itself [2]. Two widely used multipath mitigation methods, *i.e.*, high-resolution correlator (HRC) and multipath mitigation technique (MMT), and a new coupled amplitude delay lock loops (CADLL) method, wich is based on multipath signal amplitude, code phase, and carrier phase, are evaluated in [3]. They may fail under dynamic multipath scenario or when multipath is stronger than line-of-sight (LOS). Except for GPS, DR that employs vehicle kinematic model and incremental measurements of wheel encoder is often viewed to play a crucial role in precise short-term navigation of driverless cars [4]. As one of the autonomous relative navigations, the DR technique is capable of continually providing position information. A major disadvantage of using DR for navigation is that they typically suffer from accumulated error because of wheel slippage and wheel imperfection [5]. Actually, localization accuracy can maintain only within a very short range. As a result, substantial efforts have been made to improve long-term precision and robustness through slip estimation [6].

Several complementary navigation systems, including GPS, IMU, and DR, are usually combined through a variety of information fusion methods, typically such as Kalman filter (KF) [7]. In fact, GPS or GPS–IMU can provide absolute position and orientation, even if it contains discontinuous data and/or random drifts. Contrarily, as a local navigation system, DR is able to conduct accurate localization within a certain distance or duration. However, position errors will be accumulated with increase of distance. Undoubtedly, integration of GPS–IMU and DR is a natural selection to accurately navigate driverless car. In the last few decades, a lot of multimodal data fusion methods for meeting reliable, robust, and decimeter-level requirements for driverless cars, e.g., the extended Kalman filter (EKF) and the unscented Kalman filter (UKF), has emerged. The EKF simplifies nonlinear filtering and is used for state estimation in [8,9,10,11,12,13,14,15,16,17]. Among this literature, several types of additional sources of information, including on-board motion sensors, cameras or LiDAR vision systems, and road map databases, are adopted to compensate for EKF-based navigation systems. References [8,9,10,11] improve accuracy of localization by integration of different navigation systems such as IMU, GPS, and DR. Ma *et al.* [12] combine stereo-camera sensor, IMU, and leg odometry by virtue of EKF. In [13,14], both accurate digital map and camera are integrated to improve location accuracy. Moreover, four EKF-based state estimation architectures are evaluated in [15], including nonlinear model (NLM) [16] and error model (ERM) [17], each with/without a complementary filter [18,19]. The experimental results show that NLM with a complementary filter has superior localization performance, which will be adopted to make comparison with our model in Section 3. Unlike the EKF, the UKF employs unscented transform to address approximation issues of the EKF, which is also extensively exploited in multimodal data fusions [20,21,22]. Actually, there still exist some problems even if the above two kinds of methods have been widely applied. The deficiencies of the KFs including EKF and UKF were specifically pointed out in [23]. For example, considering that there are uncertainties or unknown statistical characteristics for process and/or measurement noises, it is very hard to perform reliable multimodal data fusion. Hence, the above-mentioned fusion methods are not sufficient to establish robust and accurate state estimation. To the best of our knowledge, there have been no reports on multimodal data fusion methods based on a set of predictive models and occupancy grid constraints.

In this paper, we propose a novel data fusion method for precise localization problem of driverless car using a set of ARMA predictive models and occupancy grid constraints. It is only based on on-board GPS–IMU and DR navigation data. First, a set of ARMA models with different structural orders are used for concurrent predictions, avoiding prior selection of the order of ARMA models. Second, both grid constraints and spatial consensus check on all predictive data and current measurements are conducted to remove outliers, resulting in stationary stochastic process. Third, the standard deviation of fused data can be controlled by grid size. Finally, the extensive experimental results achieved on field tests under real urban scenarios show that the proposed multimodal data fusion method can not only smooth small jumps in bias due to satellite signal occlusion or multipath but decrease accumulated location errors caused by DR. Most importantly, the localization precision of our method outperforms existing state-of-the-art methods in terms of the identical test dataset.

This paper is organized as follows. Section 2 proposes our novel data fusion method. The experimental results and performance evaluation are provided in Section 3. Section 4 draws conclusions.

## 2. Accurate GPS–IMU/DR Data Fusion Method

### 2.1. Occupancy Grid Constraints-Based Local Navigation

Suppose that vehicle displacement at a sampling period provided by GPS–IMU integrated navigation system or DR system is denoted as ${\left[\Delta x_{k},\Delta y_{k}\right]}^{T}$, *k =* 0,1,2⋅⋅⋅⋅. Under normal circumstances, the time series $\left\{\Delta x_{1},\Delta x_{2},\cdot \cdot \cdot ,\Delta x_{k},\cdot \cdot \cdot \right\}$ and $\left\{\Delta y_{1},\Delta y_{2},\cdot \cdot \cdot ,\Delta y_{k},\cdot \cdot \cdot \right\}$ are considered as a collection of stationary stochastic processes, which implies that current state is only dependent on previous one-step or multi-step states without nonstationarity. In this paper, we use a couple of ARMAs for modeling such covariance stationary time series data. Notice that we adopt position increments ${\left[\Delta x,\Delta y\right]}^{T}$ instead of absolute positions [*x*, *y*]^T^ so as to avoid multilinear problems. In general, the selection of ARMA model order is viewed as the first step prior to parameter estimation. A comprehensive survey on methods for determining the order of ARMA can also see [24]. In the popular traditional methods, optimal model could be found on the basis of certain criterions after completing estimates of model parameters, e.g., final prediction error (FPE) [25], Akaike information criterion (AIC) [26], and minimum description length (MDL) [27,28]. In [29], eigenvectors of covariance matrix of input data rather than parameter estimation are employed to determine the model order. Using Bayesian framework, a new method for jointly estimating model order and parameters is presented in [30]. In fact, there do not exist any generic methods on the best order selection problem, although improvements have been proceeding [31], among which some policy is used to be closer to real structural model at the cost of computational complexity. Thus, the determination of model order is really regarded as one of the most difficulties. In this paper, we present a novel method that has no requirements for determining model order, where a set of ARMA models with multiple different orders are utilized for position predictions that are eventually evaluated and screened by occupancy grid constraints and spatial consensus check, together with current measurements. This leads to reasonable selection of the best structural parameter.

**Figure 1:**
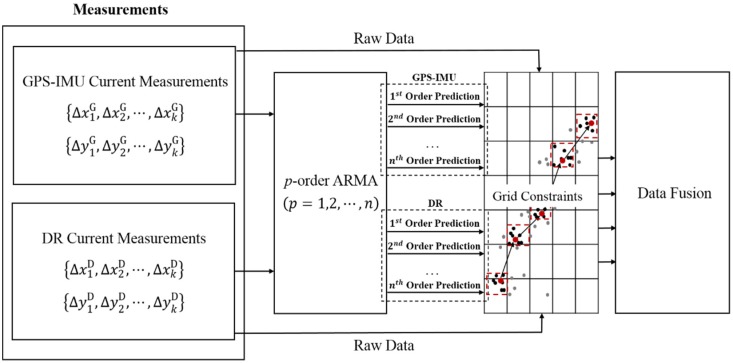
The flowchart of the proposed data fusion method.

The flowchart of the proposed multimodal data fusion method is shown in Figure 1, where $\left\{\Delta x_{1}^{G},\Delta x_{2}^{G},\cdot \cdot \cdot ,\Delta x_{k}^{G}\right\}$ and $\left\{\Delta y_{1}^{G},\Delta y_{2}^{G},\cdot \cdot \cdot ,\Delta y_{k}^{G}\right\}$ represent the eastern and northern displacements delivered by GPS–IMU, respectively. Similarly, $\left\{\Delta x_{1}^{D},\Delta x_{2}^{D},\cdot \cdot \cdot ,\Delta x_{k}^{D}\right\}$ and $\left\{\Delta y_{1}^{D},\Delta y_{2}^{D},\cdot \cdot \cdot ,\Delta y_{k}^{D}\right\}$ denote the eastern and northern displacements given by DR, respectively. The two groups of time series are used to predict current positions though a set of ARMA models with different orders. Considering that ARMA models contains multiple orders, as described as the *p*-order ARMA (*p* = 1,2,⋅⋅⋅,*n*) in Figure 1, a total of 2*n* predictions can be yielded. With the addition of current measurements by GPS–IMU and DR, we make further use of grid constraints and spatial consensus check to have removal of outliers, in order to fulfill data fusion for resulting stationary stochastic processes.

### 2.2. Prediction Using a Set of ARMA Models with Different Orders

After collecting raw data from GPS–IMU and DR, it is required to establish a set of ARMA models with multiple structural orders for prediction of localization. In this paper, we adopt 1st order, 2nd order, and 3rd order ARMA predictive models, respectively. Without loss of generality, ARMA predictive model for GPS–IMU data can be expressed as,

(1a) $$\Delta x_{k}^{G}=\Phi_{1}\Delta y_{k-1}^{G}+\Phi_{2}\Delta y_{k-2}^{G}+\cdot \cdot \cdot +\Phi_{p}\Delta y_{k-p}^{G}+\theta_{1}\Delta x_{k-1}^{G}+\theta_{2}\Delta x_{k-2}^{G}+\cdot \cdot \cdot +\theta_{p}\Delta x_{k-p}^{G}$$

(1b) $$\Delta y_{k}^{G}={\Phi '}_{1}\Delta y_{k-1}^{G}+{\Phi '}_{2}\Delta y_{k-2}^{G}+\cdot \cdot \cdot +{\Phi '}_{p}\Delta y_{k-p}^{G}+{\theta '}_{1}\Delta x_{k-1}^{G}+{\theta '}_{2}\Delta x_{k-2}^{G}+\cdot \cdot \cdot +{\theta '}_{p}\Delta x_{k-p}^{G}+{\varepsilon '}_{k}$$

where $\left(\Delta x_{k}^{G},\Delta y_{k}^{G}\right)$ denotes prediction at time step *k* using previous *p* position increments or displacements $\delta_{k,p}={\left[\Delta y_{k-1}^{G},\Delta y_{k-2}^{G},\cdot \cdot \cdot ,\Delta y_{k-p}^{G},\Delta x_{k-1}^{G},\Delta x_{k-2}^{G},\cdot \cdot \cdot ,\Delta x_{k-p}^{G}\right]}^{T}$, *p* = 1,2,..., n (e.g., *n* = 3). Specifically, $\theta ={\left[\Phi_{1},\Phi_{2},\cdot \cdot \cdot \Phi_{p},\theta_{1},\theta_{2},\cdot \cdot \cdot ,\theta_{p}\right]}^{T}$ or $\theta '={\left[\Phi {'}_{1},\Phi {'}_{2},\cdot \cdot \cdot \Phi {'}_{p},\theta {'}_{1},\theta {'}_{2},\cdot \cdot \cdot ,\theta {'}_{p}\right]}^{T}$ represents the 2*p*-dimensional vector constituted by unknown parameters, $\varepsilon_{k}$ or $\varepsilon {'}_{k}$ indicates noise, and the superscript “G” denotes position increments from GPS–IMU data. If it is from DR, the superscript is expressed by “D”. Assume that $\varepsilon_{k}$ is statistically independent and distributed with Gaussian distribution with zero mean and variance of $\sigma^{2}$. Consequently, likelihood estimation of parameter vector *θ* is stated as follows:

The noise probability density function $\varepsilon_{k}$ is given by

(2) $$p(\varepsilon_{k})=\frac{1}{{\left(2\pi \sigma^{2}\right)}^{1/2}}\exp[-\frac{{\left(\varepsilon_{k}\right)}^{2}}{2\sigma^{2}}]$$

Apparently, we have

(3) $$p(\Delta y_{k}^{G}|\delta_{k,p},\theta )=\frac{1}{{\left(2\pi \sigma^{2}\right)}^{1/2}}\exp[-\frac{{\left(\Delta y_{k}^{G}-\theta^{T}\delta_{k,p}\right)}^{2}}{2\sigma^{2}}]$$

The left side of Equation (3) indicates that this is the probability distribution function of $\Delta y_{k}^{G}$, *i.e.*, $p(\Delta y_{k}^{G}|\delta_{k,p},\theta ),\theta \sim N(\theta^{T}\delta_{k,p},\sigma^{2})$.

Note that the probability of data is a function of *Y*, which contains all the $\Delta y_{k}^{G}$ for a fixed value of θ, *i.e.*, $Y={\left[\Delta y_{1}^{G},\Delta y_{2}^{G},\cdot \cdot \cdot ,\Delta y_{k}^{G}\right]}^{T}$. If it is considered as a function of θ, then the likelihood function can be described below,

(4) L(θ)=L(θ;Y,δ)=p(Y|δ,θ)

where δ indicates matrix that contains all the $\delta_{k,p}$.

Considering that $\Delta y_{k}^{G}$ is statistically independent, the likelihood function is rewritten as

(5) $$L(\theta )=\prod_{k=1}^{m}p(\Delta y_{k}^{G}|\delta_{k,p},\theta )=\prod_{k=1}^{m}\frac{1}{{\left(2\pi \sigma^{2}\right)}^{1/2}}\exp[-\frac{{\left(\Delta y_{k}^{G}-\theta^{T}\delta_{k.p}\right)}^{2}}{2\sigma^{2}}]$$

As a result, θ should be estimated so as to make data have high probability. Instead of directly maximizing L(θ), we can also make maximization of any strictly increasing function of L(θ) such as log-likelihood function logL(θ), *i.e.*,

(6) $$\log L(\theta )=\sum_{k=1}^{m}\log p(\Delta y_{k}^{G}|\delta_{k,p},\theta )$$

By maximizing the logL(θ) [26], we can optimally find the set of parameters θ of ARMA models. Correspondingly, unknown parameters of 1st order, 2nd order, and 3rd order ARMA predictive models can on-line be estimated.

### 2.3. Accurate Data Fusion Method

At time step *k*, two current measurements from GPS–IMU and DR, respectively, together with six predictions delivered by the above-mentioned ARMA predictive models with 1st order, 2nd order, and 3rd order, are all projected onto identical occupancy grid map for data fusion. Owing to the fact that there always exist measurement noises and prediction errors caused probably by incorrect model orders and nonlinearities, this paper presents a novel policy of eliminating outliers through occupancy grid constraints and spatial consensus check (shown in Algorithm l). In the accurate data fusion method, we only choose those grid cells that contain the majority of predictions and current measurements, including that of falling on the frontier. With the grids map, the number of points falling into the same grid is counted. In this case, dense data of solely falling into an occupancy grid of *H × W* are retained as inliers, while sparse data scattered in other grids are classified as outliers. Our empirical data illustrates that the selection of both *H* and *W* equal to 0.2 m leads to the best performance. After eliminating outliers, we conduct refinement of inliers through Algorithm 1. Specifically, by iteratively filtering noise inside grids, the best localization of centroids could be estimated among inliers.

**Figure 2:**
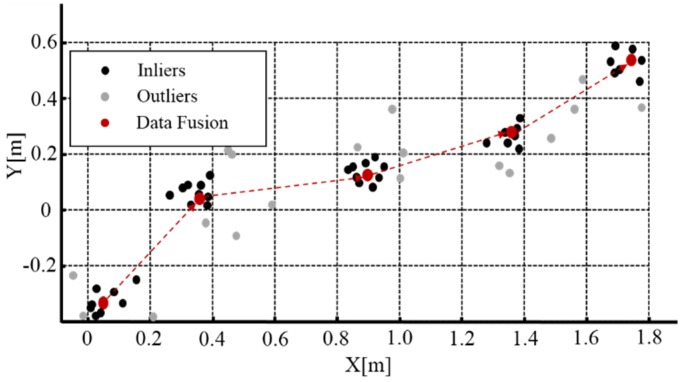
Occupancy grid based data fusion method for GPS–IMU and DR.

Figure 2 shows the grid-based data fusion scheme, where the current navigation data measured by GPS–IMU and DR, as well as the predictions obtained using multiple ARMA models presented in Section 2.2 are all indicated in black points, while the grey points stand for the outliers that should be eliminated and the red points denote the data fusion results evaluated in accordance with our method.

**Algorithm 1.** Spatial Consensus Check**Input**: $\left\{\left(\Delta x_{k-n}^{G},\Delta y_{k-n}^{G}\right),\;\cdots ,\left(\Delta x_{k}^{G},\Delta y_{k}^{G}\right),\left(\Delta x_{k-n}^{D},\Delta y_{k-n}^{D}\right),\;\cdots ,\left(\Delta x_{k}^{D},\Delta y_{k}^{D}\right)\right\}$**Output**: $\left\{\left(\Delta {\bar{x}}_{k},\;\Delta {\bar{y}}_{k}\right)\right\}$**for**
*i* = 1 **to**
*n*
**do**
$$\Delta {\tilde{x}}_{ki}^{G}\;=\;\emptyset_{xi1}^{G}\Delta x_{k-1}^{G}+\cdots +\emptyset_{xii}^{G}\Delta x_{k-i}^{G}+\theta_{xi1}^{G}\Delta y_{k-1}^{G}+\cdots +\theta_{xii}^{G}\Delta y_{k-i}^{G}$$
$$\Delta {\tilde{x}}_{ki}^{D}\;=\;\emptyset_{xi1}^{D}\Delta x_{k-1}^{D}+\cdots +\emptyset_{xii}^{D}\Delta x_{k-i}^{D}+\theta_{xi1}^{D}\Delta y_{k-1}^{D}+\cdots +\theta_{xii}^{D}\Delta y_{k-i}^{D}$$
$$\Delta {\tilde{y}}_{ki}^{G}\;=\;\emptyset_{yi1}^{G}\Delta x_{k-1}^{G}+\cdots +\emptyset_{yii}^{G}\Delta x_{k-i}^{G}+\theta_{yi1}^{G}\Delta y_{k-1}^{G}+\cdots +\theta_{yii}^{G}\Delta y_{k-i}^{G}$$
$$\Delta {\tilde{y}}_{ki}^{D}\;=\;\emptyset_{yi1}^{D}\Delta x_{k-1}^{D}+\cdots +\emptyset_{yii}^{D}\Delta x_{k-i}^{D}+\theta_{yi1}^{D}\Delta y_{k-1}^{D}+\cdots +\theta_{yii}^{D}\Delta y_{k-i}^{D}$$**end**$n_{1}^{'}\;=\;n_{2}^{'}\;=n$**for**
*i* = 1 **to**
*Max_iters*
**do**
$$\Delta {\bar{x}}_{k}\;=\;\frac{\Delta {\tilde{x}}_{k1}^{G}+\cdots +\Delta {\tilde{x}}_{kn_{1}^{'}}^{G}+\Delta {\tilde{x}}_{k1}^{D}+\cdots +\Delta {\tilde{x}}_{kn_{2}^{'}}^{D}+\Delta x_{k}^{G}+\Delta x_{k}^{D}}{n_{1}^{'}\;+\;n_{2}^{'}+2}$$
$$\Delta {\bar{y}}_{k}\;=\;\frac{\Delta {\tilde{y}}_{k1}^{G}+\cdots +\Delta {\tilde{y}}_{kn_{1}^{'}}^{G}+\Delta {\tilde{y}}_{k1}^{D}+\cdots +\Delta {\tilde{y}}_{kn_{2}^{'}}^{D}+\Delta y_{k}^{G}+\Delta y_{k}^{D}}{n_{1}^{'}\;+\;n_{2}^{'}+2}$$  Compute the distance $\vec{L}$ of all $n_{1}^{'}\;+\;n_{2}^{'}+2$ points to centroid points.  For a point $\left(\Delta x^{\prime },\;\Delta y^{\prime }\right)$, denotes $\vec{L^{\prime }}\;=\;\frac{\mathrm{sum}\left(\vec{L}\right)\;-\;L^{\prime }}{\left(n_{1}^{'}\;+\;n_{2}^{'}+2\right)\;-\;1}$  **if**
$\frac{\left\|(\Delta x^{\prime },\;\Delta y^{\prime })\right\|\;-\;\left(\Delta \bar{x},\;\Delta \bar{y}\right)}{\bar{L^{\prime }}}>threshold$, **then**    Remove this point.    Update $n_{1}^{'}\;$ or $n_{2}^{'}$.  **end****end**

After iteratively filtering noise inside grids, we get the final increments at time step *k* by averaging all the inliers.

## 3. Experimental Results

**Figure 3:**
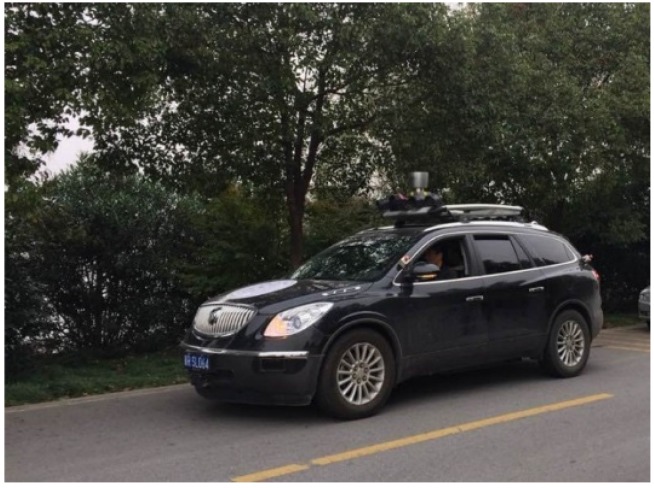
Driverless car developed by Tsinghua University.

To evaluate navigation performance of our method, we performed extensive on-site navigation experiments on our driverless car shown in Figure 3. In this section, we first make analysis of raw navigation data given by GPS–IMU and DR. Along the ground truths of autonomously driving trajectories, we then investigated position errors for stand-alone GPS–IMU, DR and our data fusion method. The ground truth is found through tight integration of NovAtel GPS receiver and IMU in the open air when the GPS mode is RTK. In addition, on the basis of datasets provided by [15], we conducted comparative study of the proposed method with state-of-the arts such as state dependent Riccati equation (SDRE) navigation filtering [15,16], which is an alternative to the EKF. Evaluation of four Kalman filtering based state estimation architectures is given in [15], which demonstrates that the NLM outperforms the other three architectures in the experiments provided in [15]. Hence, our method will be made comparison with SDRE in Section 3.2. Finally, the multimodal data fusion results in the presence of interrupted GPS–IMU signals will be further discussed.

### 3.1. Analysis of Raw Navigation Data

Let us first examine the characteristics of raw navigation data collected by GPS–IMU that is installed on our driverless car. We kept a record of position and orientation data over 10 min when the driverless car parking at three different locations (also see Table 1). It is observed from Table 1 that there is significant change in *x*-coordinates at the identical location #1 over 10 min, the maximum value of which is up to 6.102 m with the standard deviation of 1.428. Note that location #1 is just under overpass with heavy traffic flow. In this situation, GPS–IMU data become instable due to severe satellite signal occlusion and multipath effects. However, position data of (*x,y*) recorded at locations #2 and #3 always remain very stable because they are sampled in an open site.

Meanwhile, our driverless car is equipped with two wheel encoders (Nemicon SBH-1024-2MD), which can precisely produce the rolling turns of wheels. We then calculate trajectory distances of the two wheels and take the averaging of them as DR outputs. Unlike GPS–IMU data, DR data look like rather stationary but have unusual accumulated errors.

### 3.2. Comparative Study

**Figure 4:**
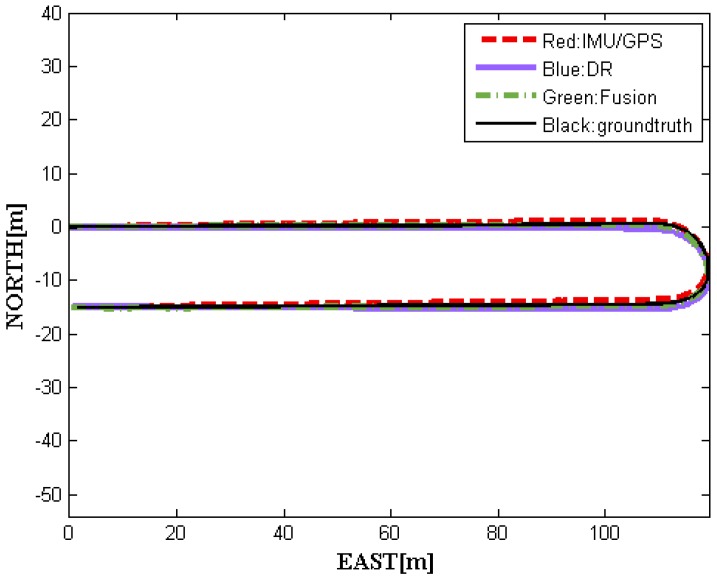
The four global trajectories recorded by vehicle.

**Figure 5:**
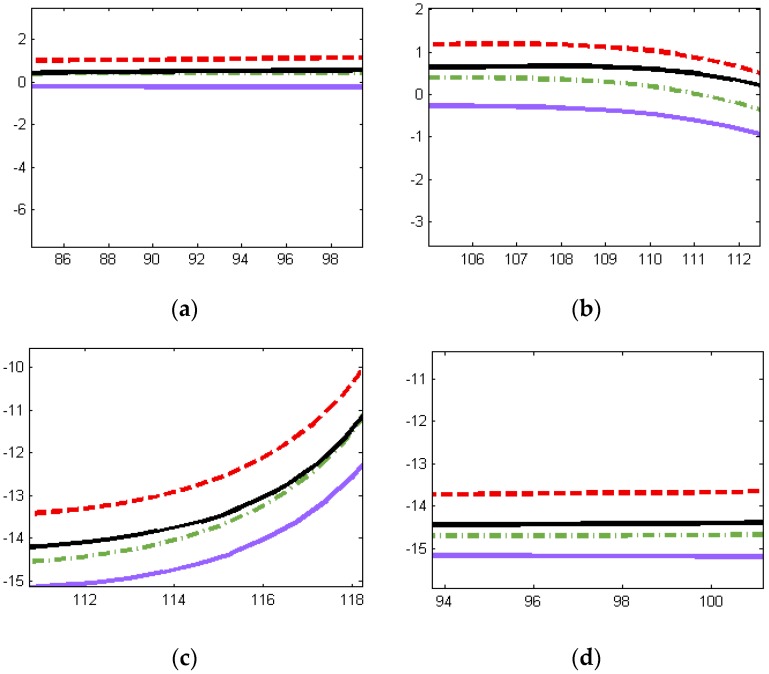
Locally enlarged trajectories. (**a**) In the vicinity of the beginning; (**b**) In the vicinity of the first curved segmentation; (**c**) In the vicinity of the second curved segmentation; (**d**) In the vicinity of the destination.

Figure 4 shows four moving trajectories of driverless cars on a real route containing curved roads, including GPS–IMU measurements, DR outputs, and data fusion obtained using our methods, as well as the ground truth. Apparently, the former three trajectories are roughly consistent with the ground truth. To gain more clear insights about performance, we have four local trajectories enlarged, as shown in Figure 5. Notice that it has the same legend as in Figure 4.

**Figure 6:**
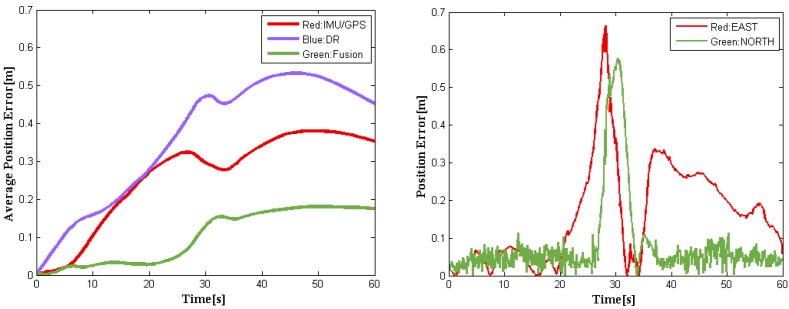
Average position error curves (**Left**) evaluated using IMU–GPS, DR, and our data fusion method. Errors in position are obtained as the norm of difference between the ground truth and the fusion data at each time step (**Right**).

Figure 5 demonstrates that the fusion results we achieved are almost between the GPS–IMU raw data and DR data, keeping smooth and steady, even in curved segmentation. Compared to the ground truth, our data fusion results are much closer to the actual localization physically going with the driverless car. To demonstrate the relative localization performances, Figure 6 (left) shows the average position error curves of the three trajectories derived from GPS–IMU, DR, and our method, respectively. The final average position error is only 0.18 m for the fusion data when the DR has already an error of 0.49 m. The difference between the fusion data and the reference is also plotted in Figure 6 (right). The left curves in Figure 6 indicate average position errors. In other words, position errors will be averaged over time, which can clearly reflect accumulation of errors. However, the right ones represent position errors at a specific time in our experiment. The overall position root–mean-square (RMS) errors are calculated to be 0.47 m for GPS–IMU 0.58 m for DR, and 0.25 m for the proposed method, respectively. Actually, our fusion method still needs to be further improved when turning-about, even if the fusion results are much better than that of either GPS–IMU or DR data for straight or curved roads. It is probably caused by big azimuth error of DR when crossing intersection.

**Figure 7:**
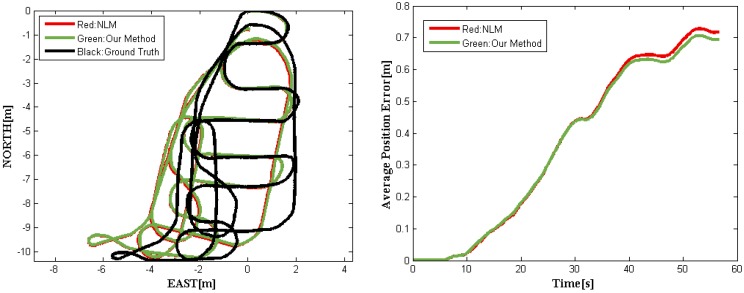
Trajectories obtained by our method and with NLM [15] (**Left**). The evolving of average position errors over time (**Right**).

**Figure 8:**
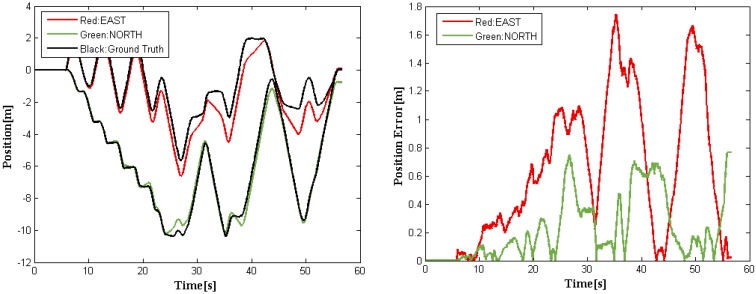
The corrected position (**Left**) corresponding to the trajectory in Figure 7. Errors in position are obtained as the norm of difference between the ground truth and our fusion data at each time-step (**Right**).

In general, the EKF is proven to work very well among all data fusion methods. Using the datasets on real road and the code implementation provided by [15], we carried out a comparative study of our method with NLM (SDRE filter). The results are shown in Figure 7 and Figure 8. Figure 7 shows the trajectories obtained by different fusion methods and the average position errors of the proposed method and NLM when compared with the ground truth. Figure 8 depicts our fusion data with the reference values. Note that the ground truth was obtained using top-down camera tracking of the driverless car. The precision of visual tracking system was experimentally determined to be 15 cm ± 12 cm within 15 m × 10 m outdoor area. The red solid curve represents the experimental results achieved with NLM, while our position error is marked in green solid line. It is easy to see from Figure 7 that our method outperforms NLM. The position RMS errors of the above-mentioned methods are listed in Table 2.

From Figure 7 and Figure 8 and Table 2, it is readily observed that our method performs better than the NLM in [15], which outperforms classical NLM without complementary [16] and ERM [17]. Additionally, we conduct an additional analysis of computational load for the above methods. Note that the cycle duration of the NLM is estimated using the simulation (MATLAB) [15], while that of our new method is evaluated through the on-board system of our driverless car implemented by C++. And the results are listed in Table 3. Although the comparison is unfair, it illustrates that the proposed method does have superior performance in terms of accuracy and computational load.

### 3.3. Data Fusion Results with Interrupted GPS–IMU Signal

When satellite signal occlusion or multipath error occurs, position estimates delivered by GPS–IMU might be severely biased occasionally. Figure 8 shows the experimental results obtained using our fusion method, which indicates that the proposed method is capable of providing steady and continual vehicle trajectories even if there is bias or even big interruption in GPS–IMU signals.

**Figure 9:**
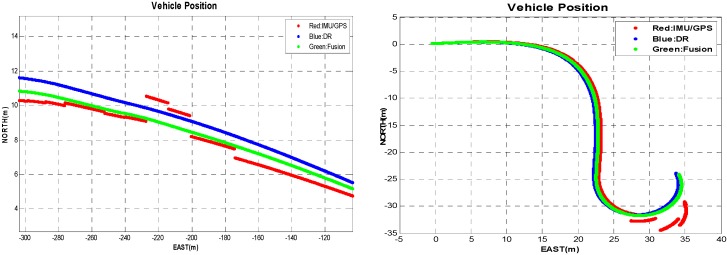
The data fusion results when interrupted GPS–IMU signals are present for straight roads (**Left**) and curved roads (**Right**).

It is readily observed from Figure 9 that there indeed exist small discontinuity jumps in bias due to satellite signal occlusion or multipath, as indicated in the red marked GPS–IMU raw data, which is occasionally even up to 2 m in bias and intolerable for navigation systems of driverless cars. However, our new data fusion method makes vehicle trajectories perfectly smooth, as shown in curves marked in green and the localization accuracy is almost unaffected by a jump in GPS–IMU data. In fact, the proposed adaptive data fusion method is practically applied in THU-IV2 driverless cars developed by ourselves.

## 4. Conclusions

In this paper, we propose a new multimodal data fusion method for accurate navigation of driverless cars based on a set of predictive models and occupancy grid constraints. The novelty of our method includes: (1) we employ a set of ARMA models with different structural orders to concurrently make location predictions, avoiding subjectively determining the order of ARMA models; (2) both grid constraints and spatial consensus check are presented to have removal of outliers, in order to generate stationary stochastic process; and (3) the standard deviation of data fusion can be controlled by size of grid in advance. To evaluate navigation performance of the proposed method, we conduct a considerable amount of on-site experiments. The experimental results demonstrate that our method can not only smooth small jumps in bias due to satellite signal occlusion or multipath but also achieve promising localization fusion precision. Although accumulated position errors caused by DR are significantly reduced, there still remains a certain value with the increase of distance in a sense. In particular, degradation in fusion accuracy will occur when GPS–IMU retains discontinuity jumps in bias for a longer period of time. In this case, a policy on blocking GPS–IMU data sources should be adopted.

## Figures and Tables

**Table 1:** Raw records of position data over 10 min.

**(*X*[m], *Y*[m])**	#**1**	#**2**	#**3**
	The driverless car parked at three different locations numbered #1, #2, #3		
(*X*_min, *Y*_min)	(−2521.839, 1990.442)	(−215.399, 190.123)	(−0.013, −0.001)
(*X*_max, *Y*_max)	(−2515.737, 1990.554)	(−215.393, 190.134)	(0.099,0.397)
(*X*_average, *Y*_average)	(−2516.651, 1990.461)	(−215.396, 190.129)	(0.038, 0.255)
(*X*_stdev, *Y*_stdev)	(1.428, 0.022)	(0.001,0.002)	(0.037, 0.128)

**Table 2:** Performance comparison of our method with the nonlinear model (NLM) in [15].

Approach	North Position RMSE (m)	East Position RMSE (m)	Position RMSE (m)
NLM[15]	0.8241	0.3268	0.8865
Our Method	0.7973	0.3171	0.8581

**Table 3:** Analysis of computational load.

	NLM	NLM + CF	Our Method
Cycle duration (ms)	0.8	1.0	0.005
